# The double-edged sword effect of ethical leadership on constructive deviance: An integrated model of two approaches based on organizational identification and normative conflict

**DOI:** 10.3389/fpsyg.2022.892395

**Published:** 2022-07-19

**Authors:** Lixia Niu, Wende Xia, Yong Liu

**Affiliations:** ^1^College of Business Management, Liaoning Technical University, Huludao, China; ^2^School of Economic and Management, Southeast University, Nanjing, China

**Keywords:** constructive deviance, ethical leadership, organizational identification, normative conflict, double-edged sword effect

## Abstract

Based on the normative conflict model, this study proposes a dual-pathway model that is constituted of organizational identification and normative conflict, and examines the double-edged sword effect of ethical leadership on subordinates’ constructive deviance. According to the analysis of 449 questionnaires collected from Chinese employees, the results show that ethical leadership can promote employees’ constructive deviance by improving their constructive intention (Organizational identification), and it can weaken employees’ deviance motivation (normative conflict) to prevent their constructive deviance. Moreover, ethical leadership has different effects on different types of constructive deviance. This research further enriches the formation mechanism of constructive deviance and provides practical guidance to exert the effectiveness of constructive deviance in organizational management.

## Introduction

In the VUCA era, the uncertainty of the external environment and the low efficiency of the internal operation of the organization make the traditional organization management face the dilemma of simultaneous internal and external risks (Khan et al., 2021). Organizations need to make decisions quickly according to the changes in the external environment, and some inappropriate rules and regulations in organizations will restrain the behavior of organization members, delay the decision-making of the organization and seriously affect the performance of enterprises. This requires employees to break the mold and become “loyal rebel” when necessary (Dahling and Gutworth, 2017). The academic community refers to such behavior of employees as constructive deviance, which symbolizes voluntary behavior that violates the organizational norms but brings benefits to the organization and its members (Galperin, 2003). As a catalyst for organizational change, constructive deviance can further release the potential of employees’ innovation, meet their self-actualization needs and improve the competitiveness of organizations (Dahling and Gutworth, 2017; Zhao, 2019). But as the research went further, the researchers found that constructive deviance does not always play a glamorous role (Zhou and Qian, 2021). Constructive deviance has a high requirement for the initiator of the behavior, employees need to accurately judge the timing of violation and weigh the benefits. As a result, many initiators do wrong things with good intentions due to their lack of ability, which will bring negative impacts to the organization (Dahling and Gutworth, 2017). In fact, what organizations need is deliberate and constructive deviance by employees, not just reckless behavior with altruistic motives (Li and Wang, 2021; Zhou and Qian, 2021). In order to remain competitive in a complex environment, organizations must solve the problem of how to enable employees to engage in deliberate, organization-friendly “deviant” behavior without compromising their constructive willingness. Unfortunately, most of the mainstream research on constructive deviance only focuses on its positive side, even though a few researchers have theoretically proposed the negative side of constructive deviance (Li and Wang, 2021; Zhou and Qian, 2021), But how to solve the double-edged sword effect of constructive deviance is not put forward.

As the information publisher and resource distributor of the organization, the leader is an important environmental factor affecting employee’s behavior (Zhang et al., 2021). Studies have confirmed that positive leadership can stimulate employees’ constructive intention and improve their psychological security, which is an important antecedent to promoting employees’ constructive deviance, such as leader moral humility (Zhang et al., 2021), coaching leadership (Cui et al., 2022) and empowering leadership (Wu and Du, 2021). Similar to other positive leadership, ethical leadership is fair, integrity and cares about employees’ well-being, which can promote employees’ “construction intention” and bring many positive results to the organization (Brown et al., 2005; Mansur et al., 2020; Ali et al., 2022). However, the most significant difference between ethical leadership and other leadership is that ethical leadership emphasizes compliance with norms and regards compliance with organizational norms as a part of practicing morality (Lemoine et al., 2019; Keem et al., 2022). Therefore, ethical leadership not only has ethics essence, but also compliance essence, which also leads to ethical leadership tends to restrain employees’ violation of organizational norms, such as social undermining behaviors (Mostafa et al., 2020) and employee deviance (Van Gils et al., 2015). Given that constructive deviance has the dual essence of “construction intention” and “behavior violation” (Liu et al., 2020; Zhou and Qian, 2021), this study suggests that ethical leadership may be the key to helping organizations solve this problem. Specifically, the ethics essence of ethical leadership promotes the construction intention of employees, while the compliance essence increases the cost of employees’ violation of rules within the organization, thereby inhibiting the employees’ deviant motivation, and enabling the organization to gain high-quality constructive deviance without affecting the constructive intention of employees (Bush et al., 2020). In addition to theoretical analysis, different researchers have also come to contradictory conclusions when discussing the relationship between ethical leadership and pro-social rule breaking (a type of constructive deviance, Déprez et al., 2021) in empirical studies (Xu and Zhu, 2017; Gao et al., 2019). Therefore, this study suggests that ethical leadership may have a double-edged sword effect on constructive deviance. To sum up, this study believes that reorganizing the relationship between ethical leadership and constructive deviance can not only help organizations solve the double-edged sword effect of constructive deviance, but also integrate the contradictory conclusions of previous studies on the relationship between ethical leadership and constructive deviance, which has important practical and theoretical significance.

Through combing the literature on ethical leadership and constructive deviance, the potential relationship between ethical leadership and constructive deviance is preliminarily established theoretically. However, the specific path of ethical leadership influencing constructive deviance should be further explored. According to the core hypothesis of the normative conflict model, only individuals with high organizational identification and experience high normative conflict will express dissent for organizational well-being (Packer, 2008). Therefore, based on the normative conflict model, this study proposes that ethical leadership may have a double-edged sword effect on employees’ constructive deviance through influencing employees’ organizational identification and normative conflict. Specifically, from the cost–benefit perspective, constructive deviance is undoubtedly a kind of behavior with high risk and low return for employees, and employees are likely to be punished by superiors due to behavioral failure (Vadera et al., 2013; Wang, 2022). So why do employees do those arduous but fruitless things? According to the normative conflict model, organizational identification is an important motivation for employees to raise dissent, and individuals with high organizational identification will put organizational interests above personal interests (Packer and Chasteen, 2010; Packer and Miners, 2014). Literature on organizational identification also shows that organizational identification is often closely related to employees’ proactive behavior (Marstand et al., 2020; Shahjehan et al., 2020). As the spokesperson of an organization, ethical leadership’s care and respect for employees can promote employees’ identification with the organization (Zhu et al., 2015). It can also improve employees’ psychological sense of belonging by shaping a positive organizational image. Therefore, employees’ organizational identification can stimulate employees’ constructive intention, which may be the key mechanism of ethical leadership to promote their constructive deviance. So how does ethical leadership restrain constructive deviance of employees? In recent years, the relevant literature on ethical leadership generally emphasizes that ethical leadership attaches importance to organizational norms, but the specific mechanism of this essence on employee behavior is still unclear (Lemoine et al., 2019; Keem et al., 2022). Given this, this study comprehensively considers the compliance essence of ethical leadership and introduces normative conflict as the mechanism of ethical leadership to “restrain” employees’ constructive deviance based on the normative conflict model. Specifically, ethical leadership emphasizes not only abiding by the organizational expectation, but also will answer questions of the organization’s regulation and adopt reasonable opinions of employees modestly, constantly adjust and improve organizational norms, weaken employees’ normative conflict from subjective and objective perspectives. It raises the threshold of employees’ violations and then restrains the employees’ constructive deviance. However, this suppression is more like a kind of screening effect, which excludes the reckless and self-interested constructive deviance and improves the overall quality of constructive deviance.

This research aims to make the following theoretical contributions: First, based on previous studies, this study further clarifies the relationship between ethical leadership and constructive deviance. Based on ethical leadership’s dual essence of ethics and compliance, this study proposes the double-edged sword effect of ethical leadership on constructive deviance (Xu and Zhu, 2017; Gao et al., 2019). It responds to the call of previous research to explore the relationship between more leadership and constructive deviance, and enriches relevant literature on ethical leadership and constructive deviance (Wang, 2022). Secondly, this study further clarifies the negative side of constructive deviance, and put forward theoretical and practical suggestions on how to cope with the double-edged sword effect of constructive deviance. For organizations, in order to make full use of the benefits of constructive deviance and avoid its disadvantages, it is necessary to increase the cost of employee violations without damaging the employees’ constructive intention, so that employees can make rational constructive deviance after careful consideration, and ethical leadership may be a wise choice for organizations. Finally, based on the normative conflict model, this study explores the dual-pathway mechanism of organizational identification and normative conflict as ethical leadership influences employees’ constructive deviance. It not only clarifies the specific path and mechanism of ethical leadership’s effect on constructive deviance, but also brings the positive and negative effects of ethical leadership into the same frame, which further enriches the mechanism of ethical leadership.

## Theory and hypothesis development

### Ethical leadership and constructive deviance

Brown et al. (2005) defined ethical leadership as “the demonstration of normatively appropriate conduct through personal actions and interpersonal relationships, and the promotion of such conduct to followers through two-way communication, reinforcement, and decision-making.” Based on the deontological approach to morality, Bush et al. (2020) suggested that ethical leadership would exhibit promotion-oriented ethical behavior and prevention-oriented ethical behavior in daily management. The promotion-oriented ethical behavior encourages employees to engage in ethical behaviors, while the prevention-oriented ethical behavior discourages employees’ unethical behaviors. Previous studies have also confirmed that ethical leadership can promote employees’ organizational citizenship behaviors (Mansur et al., 2020) and voice behavior (Bai et al., 2017), and inhibit employees’ deviance (Van Gils et al., 2015) and unethical behaviors (Kuenzi et al., 2020). Based on previous studies, this study proposes that ethical leadership has two essential attributes: ethics and compliance. While constructive deviance has the moral duality of constructive intention and behavioral violation in essence (Liu et al., 2020; Zhou and Qian, 2021), therefore, this research speculated that ethical leadership has a double-edged sword effect on constructive deviance. Specifically, the ethics essence of ethical leadership promotes the constructive intention of employees, while the compliance essence increases the cost of employees’ violation of rules within the organization, thereby inhibiting the employees’ deviant motivation, and enabling the organization to gain high-quality constructive deviance without affecting the constructive intention of employees (Bush et al., 2020).

According to the normative conflict model, employees raise dissent to the existing norms of the group to help and improve the group they belong to (Packer, 2008). Ethical leadership can stimulate and promote such constructive intention in employees (Anser et al., 2021). Specifically, ethical leadership behaves ethically in daily work and promotes employees to internalize the moral concept of the organization by building an ethical model within the organization. When facing a moral dilemma, employees will implement the thought of ethical leadership and imitate the behavior of leaders (Mo et al., 2019). In addition, ethical leadership cares about employees’ interests and will establish trust and benign interpersonal relationships with employees, so that employees are willing to take risks for the interests of the organization (Abdullah et al., 2019; Cheng et al., 2021). Therefore, under the influence of ethical leadership and interpersonal care, employees will take the initiative to engage in constructive deviance when facing opportunities to bring benefits to the organization.

However, ethical leadership also has an inhibiting effect on constructive deviance, which will reduce employees’ intention to violate organizational norms. Different from other positive leaderships, ethical leadership attaches more importance to the organizational ethical norms and strengthens ethical norms through rewards and punishments (Lemoine et al., 2019; Keem et al., 2022). Some scholars’ studies also support this view, such as Mostafa et al. (2020) suggested that under the management of ethical leadership, there will be fewer deviant behaviors in the team than in other leadership. According to the normative conflict model, employees’ dissents are based on psychological loyalty rather than objective loyalty (Packer, 2008). In other words, employees act in the way that they think is most beneficial to the organization. However, limited by their abilities and knowledge, employees may misjudge the rationality of the organizational norms, making constructive deviance degenerate into destructive deviance (Cui et al., 2020). However, employees who do wrong things with good intentions may be regarded as challenging to organizational norms and leadership authority by ethical leadership, and thus punished by leaders (Vadera et al., 2013). In addition, the normative conflict model also suggests that when employees decide whether to engage in constructive deviance, they will conduct a cost–benefit analysis of the behavior in advance (Packer, 2008). When employees perceive that the cost of violating norms is too high, or observe that a colleague is punished by his or her supervisor for violating organizational norms, they will engage in similar behaviors less often. Therefore, in an organization managed by ethical leadership, employees may be afraid to engage in constructive deviance for fear of being punished.

Although ethical leadership has a double-edged sword effect on constructive deviance, however, this research proposes that the overall effect of ethical leadership on constructive deviance should be positive. Ethical leadership attaches importance to organizational norms, which does not mean that leaders are inflexible. For example, Xu and Zhu (2017) suggested that ethical leadership has high moral maturity and will not blindly follow norms, but will challenge wrong organizational norms when necessary, thus granting legitimacy to constructive deviance within the organization. However, unlike leaders, the constructive intention of employees often leads to destructive consequences due to their lack of ability and knowledge (Dahling and Gutworth, 2017). Therefore, while encouraging employees’ constructive deviance, ethical leadership needs to raise the threshold of employees’ deviance to screen out some immature constructive deviance and ensure the overall quality of employees’ constructive deviance. In other words, the negative effect of ethical leadership on the constructive deviance of employees is actually a positive screening effect, rather than knocking down all of them. Therefore, although ethical leadership has a simultaneous double-edged sword effect on constructive deviance, its overall impact on constructive deviance should be more beneficial than harmful.

Galperin (2012) divided constructive deviance into constructive organizational deviance (COD) and constructive interpersonal deviance (CID) according to the formality of the violated norms. This research speculates that ethical leadership may be slightly different from different types of constructive deviance. Lemoine et al. (2019) believed that the management of ethical leadership lies in compliance with organizational standards and normative expectations. When clear organizational norms exist, behaviors violating the norms will become more obvious, and the risk of COD by employees with high organizational identification will be magnified (Mostafa et al., 2020). Different from COD, CID is the criticism and correction toward organizational members’ improper behaviors. Ethical leadership is willing to listen to reasonable suggestions from employees and encourage organization members to speak freely, which can reduce interpersonal risks (Bai et al., 2017). In conclusion, the overall effect of ethical leadership on COD and CID is positive, and the effect of ethical leadership on different types of constructive deviance may be different. Therefore, the following hypotheses are proposed:

*Hypothesis 1*: Ethical leadership has a positive effect on the COD of employees.

*Hypothesis 2*: Ethical leadership has a positive effect on the CID of employees.

*Hypothesis 3*: Compared with CID, ethical leadership plays a weaker role in promoting COD.

### The mediating role of organizational identification

Organizational identification is an individual’s perception of organizational identification and emotional connection to the organization (Ashforth and Mael, 1989; Ashforth et al., 2008). According to the normative conflict model, individuals with high organizational identification do not blindly assume that organizational norms are always appropriate, but will evaluate the impact of existing norms on organizational identification and interests under the premise of considering the maximum interests of the organization (Packer, 2008). Ethical leadership can promote employees’ deeper understanding of the organization and establish employees’ emotional connection with the organization, thus improving employees’ organizational identification, and then promoting employees’ constructive deviance.

Ethical leadership can promote the formation of subordinates’ organizational identification. First of all, ethical leadership emphasizes and practices organizational norms within the organization, and helps employees to have a clearer understanding of the organization through two-way communication. In this process, organizational norms and values are gradually internalized among employees, thus promoting employees’ perception of organizational identification (Zheng et al., 2021). Secondly, ethical leadership pays attention to the well-being of employees and takes the realization of their best interests into account, which can enhance the psychological connection between employees and the organization (Brown et al., 2005; Mostafa et al., 2020). In addition, according to social identity theory, the uniqueness of organizational values and practices, organizational reputation and ingroup salience are important sources of organizational identification (Ashforth and Mael, 1989). When employees are in an organization with a good reputation and unique values, it is easier to form a sense of identification with the organization. Ethical leadership pays heed to the shaping of internal ethical values and organizational ethical practices, and guides employees’ moral behaviors in daily work, which is conducive to shaping a good organizational image. As business scandals become more and more common (Cole et al., 2021), working in such organizations can help employees better understand the uniqueness of organizational values and practices, which is conducive to the formation of organizational identification.

Organizational identification can predict the constructive deviance of employees. According to the normative conflict model, organizational identification is an important motivation to promote employee’s dissent (Packer and Miners, 2014). Specifically, when making behavioral decisions, employees with high organizational identification will consider the influence of such behavior on organizational interests and positive organizational identity (Packer, 2008). On the one hand, organizational identification can stimulate the motivation of employees to meet organizational needs and prompt employees to regard themselves and the organization as a community of interests and pay more attention to the collective interests of the organization (Buil et al., 2019; Wang and Liu, 2020). When employees identify with the organization, they will be more committed to working for the interests of the organization, and organizational identification can significantly improve employee performance and organizational citizenship behavior (Buil et al., 2019; Marstand et al., 2020). On the other hand, organizational identification can motivate employees to maintain a positive organizational identity (Shuman et al., 2018). Sillince and Golant (2018) believed that the negative evaluation of organizational stakeholders would threaten the identity of organizational members, while employees with high organizational identification would actively take actions to defend their organizational identity (Ashforth et al., 2008). For example, store employees may replace goods for customers in order to avoid negative comments on their store. Although such behaviors may bring economic losses to the organization, employees will regard them as constructive behaviors that are beneficial to the long-term development of the organization. In conclusion, ethical leadership may promote the constructive deviance of employees by promoting their constructive motivation, namely organizational identification.

The normative conflict model holds that organizational identification is an important prerequisite for employees to raise dissent. However, theories related to social identity and empirical studies generally draw opposite conclusions, that is, employees with high organizational identification tend to be loyal supporters of organizational order (Blader et al., 2017). Therefore, when employees with high organizational identification engage in constructive deviance, they will be in a state of ethical decision: break organizational norms for the benefit of the organization or follow the rules to maintain the authority of the organization? This research speculates that the formality of norms would affect this ethical decision process of employees. As mentioned above, an important source of organizational identification is the perception of organizational identification. In order to maintain their positive organizational identity, employees will try their best to reduce behaviors that break the formal norms of the organization, such as COD. In contrast, CID violates the conventional interpersonal norms rather than the formal norms of the organization, and the formal degree of norms is lower. Therefore, the following hypotheses are proposed:

*Hypothesis 4*: Ethical leadership promotes COD by positively influencing employees’ organizational identification.

*Hypothesis 5*: Ethical leadership promotes CID by positively influencing employees’ organizational identification.

*Hypothesis 6*: Compared with CID, ethical leadership plays a weaker role in promoting COD through organizational identification.

### The mediating role of normative conflict

According to the normative conflict model, individuals will experience normative conflict when they perceive differences between the actual norm of the group and some better alternative standards (Packer, 2008; Dahling and Gutworth, 2017). Warren (2003) held that constructive deviance violates organizational norms, but conforms to Hyper-norms. When individuals perceive the difference between organizational norms and hyper-norms, the normative conflict will prompt employees to engage in constructive deviance. This research speculates that ethical leadership will weaken employees’ normative conflict and inhibit their constructive deviance.

Ethical leadership can improve the objective rationality and subjective authority of organizational norms to reduce employee’s normative conflict. On the one hand, ethical leadership respects the opinions of their subordinates and takes the initiative to discuss business values and ethics with them (Brown et al., 2005). Through two-way communication with employees, humbly accepting reasonable suggestions from employees, constantly adjusting and improving organizational norms, ethical leadership objectively improves the rationality of organizational norms, and then weakens employees’ normative conflict; On the other hand, ethical leadership attaches importance to ethical norms and normative standards within an organization (Lemoine et al., 2019). Ethical leadership will instill the legitimacy and authority of the organizational norms to employees, answer their questions about the organizational norms, and urge employees to recognize the organizational norms sincerely. When there is a conflict between the organizational norms and the real situation, employees will choose organizational norms as their criteria. In addition, when employees perceive that there is room for improvement in the organization’s existing norms, but their opinions are not adopted by their superiors, they will also give priority to reflecting on whether their ability or knowledge is limited, rather than directly due to the defects in the organization’s norms. According to the normative conflict model, when experiencing highly normative conflict, group members will actively participate in behaviors against existing organizational norms (Packer, 2008). However, low normative conflict means that employees subjectively believe that there is no improvement in the existing organizational norms, and consider abiding by the organizational norms strictly as the optimal decision, any form of deviant behavior at this point will be seen as undermining the organization by the employees. In conclusion, ethical leadership inhibits constructive deviance of employees by weakening their deviant motivation, namely normative conflict.

In addition, the normative conflict model holds that different normative conflicts will occur according to the difference in violation of the norms (descriptive norms, prescriptive norms), and then leads to different types of dissents (Packer, 2008; Packer and Chasteen, 2010). This research further speculates that the dimension division of constructive deviance also conforms to this criterion. COD is an individual’s violation of the formal norms of the organization in order to improve the overall well-being of the organization, which is dissent caused by prescriptive normative conflict. CID is dissent caused by descriptive normative conflict, which violates the conventional rules established in the organization (Galperin, 2012). In fact, under the same level of normative conflict, there are great differences in the dissents caused by different types of normative conflicts in the same environment. For example, ethical leadership is open-minded and listens to reasonable opinions from employees, but does not tolerate employees’ behaviors that openly violate organizational norms (Mostafa et al., 2020). According to the normative conflict model, when employees decide whether to engage in dissent, they will conduct a cost–benefit analysis of the behavior in advance (Packer, 2008). In essence, the two types of constructive deviance break organizational norms for organizational well-being, and there is no significant difference in benefits, but in cost, the cost of breaking formal organizational norms is much higher than that of breaking informal interpersonal norms. Therefore, this research argues that ethical leadership has a greater negative effect on COD than CID through normative conflict. Therefore, the following hypotheses are proposed:

*Hypothesis 7*: Ethical leadership inhibits COD by negatively influencing employees’ normative conflict.

*Hypothesis 8*: Ethical leadership inhibits CID by negatively influencing employees’ normative conflict.

*Hypothesis 9*: Compared with CID, ethical leadership has a stronger inhibitive effect on COD through normative conflict.

**Figure 1:**
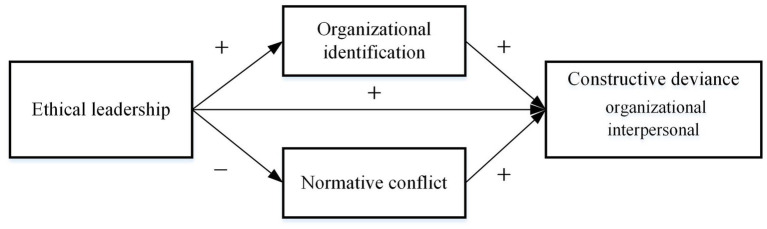
Theoretical model.

Based on the above analysis, the theoretical model of this study is shown in Figure 1.

## Materials and methods

### Sample and data collection

The samples for this study came from 30 enterprises in Liaoning, Beijing, and Zhejiang of China, mainly including construction, banking, information technology and other industries. Based on the preliminary research of the research group, this study selected 19 Liaoning local enterprises that had pleasant cooperation experiences as the investigated enterprises. In addition, in order to increase the sample size and reduce the impact of regional differences, the research group contacted 11 enterprises located in Beijing, Zhejiang through alumni relations. With the support of the enterprise’s leader, the research group contacted the human resources department and asked for the name list of the grass-roots staff (including name only). The research group set an appropriate sampling ratio according to the size of the company, and selected the sample frame of this study according to the principle of random sampling including a total of 561 respondents. In order to ensure the quality of the questionnaire, a preliminary survey was conducted before the formal survey, and the wording and presentation of some questions in the questionnaire were adjusted appropriately according to the results of the preliminary survey. Questionnaires were collected anonymously, and the researchers highly emphasized the confidentiality of data. In order to ensure the sample size is sufficient, questionnaires were collected online and offline and lasted for about 2 months from the beginning of September 2021 to the end of October 2021. First of all, the research group sent questionnaires to some local enterprises in Liaoning province on the spot, and collected 347 paper questionnaires on the spot after the questions were answered. Second, team members contacted enterprise managers in Beijing, and Zhejiang, with the consent of the manager, the research group entrusted them to distribute the electronic questionnaire to their subordinates through online social networking tools (e.g., WeChat). In order to improve the quality of the electronic questionnaire and the participants’ enthusiasm, every participant can be rewarded with petty cash after check. As a result, a total of 212 electronic questionnaires were collected. Finally, a total of 449 valid questionnaires were obtained with an effective recovery rate of 80.3% after screening the 559 collected questionnaires (e.g., excluding invalid questionnaires with many defects, regular answers, failing to pass the test of careful answers, too long or too short answers et al.) The basic situation of valid samples is as follows: the proportion of females is slightly higher than that of males, accounting for 52.8%; the age was mainly 36–45 years old, accounting for 56.1%; most of them were married, accounting for 53.5%. Most of them had a bachelor degree, accounting for 72.4%; 69.3% of them have worked for less than 3 years and 3–5 years. Demographic characteristics are described in Table 1.

**Table 1:** Sample characteristics (*n* = 449).

**Variables**	**Categories**	***N***	**%**
Gender	Male	212	47.2
	Female	237	52.8
Age	18–25 Years	135	30.1
	26–35 Years	252	56.1
	36–45 Years	46	10.2
	46–55 Years	12	2.7
	56 Years and above	4	0.9
Marital status	Married	240	53.5
	Unmarried	209	46.5
Tenure	3 Years and below	171	38.1
	3–5 Years	140	31.2
	6–10 Years	103	22.9
	11 Years and above	35	7.8
Education	High School and above	14	3.1
	Junior College	43	9.6
	Bachelor’s Degree	325	72.4
	Master’s Degree and Above	67	14.9

### Measures

In order to ensure the reliability and validity of the scale, all the scales in this research are mature scales published in journals and verified by many empirical studies in the Chinese context. In addition, this research strictly followed the “translation-back” procedure and invited experts to review the translated scale, so as to ensure that the Chinese scale could accurately restore the meaning of the original English scale (Cheng et al., 2020). The Likert 5-point scoring method is used for all scales, with 1 ~ 5 indicating from “strongly disagree” to “strongly agree.”

### Ethical leadership

The measurement of this variable adopts the scale developed by Brown et al. (2005), which includes 10 items such as “my leader will Discipline employees who violate ethical norms.” In this research, the Cronbach’s α was 0.905.

### Constructive deviance

This variable is measured by the scale developed by Galperin (2012), which includes two dimensions of constructive organizational deviance (COD) and constructive interpersonal deviance (CID). 5 items of COD, representing items such as “I will violate organizational rules and procedures in order to solve problems,” Cronbach’s α was 0.848. 4 items of CID, representing items such as “in order to promote the development of the organization, I will point out colleagues’ mistakes in work,” Cronbach ‘α was 0.851.

### Organizational identification

This variable was measured by a 6 items scale developed by Mael and Ashforth (1992), which represented items such as “when someone criticizes my organization, I feel insulted.” In this research, the Cronbach’s α was 0.881.

### Normative conflict

The measurement of this variable adopts the 8 items scale developed by Dahling and Gutworth (2017), which represents items such as “This company will never reach its true potential until it changes its practices.” In this research, the Cronbach’s α was 0.924.

### Control variables

Referring to previous studies (Dahling and Gutworth, 2017; Wang and Liu, 2020; Zhang et al., 2021), demographic variables such as gender, marital status, years of working in the unit and education level were selected as control variables in this research.

## Results

### Descriptive statistics

SPSS 24 was used for descriptive statistical analysis in this research, and the mean, standard deviation and correlation coefficient of each variable are shown in Table 2. As can be seen from the table, ethical leadership is significantly positively correlated with COD (*r* = 0.133, *p* < 0.01), CID (*r* = 0.168, *p* < 0.01), and organizational identification (*r* = 0.550, *p* < 0.01). It is negatively correlated with the normative conflict (*r* = − 0.407, *p* < 0. 01). Organizational identification is positively correlated with COD (*r* = 0.127, *p* < 0.01) and CID (*r* = 0.222, *p* < 0.01). Normative conflict is positively correlated with COD (*r* = 0. 204, *p* < 0. 01) and is no significant correlation with CID due to the possible masking effect (Wen and Ye, 2014).

**Table 2:** Means, standard deviations, and correlation coefficients (*n* = 449).

S. No.		1	2	3	4	5	6	7	8	9	10
1.	Gender	1									
2.	Age	−0.117^*^	1								
3.	MS	0.057	−0.596^**^	1							
4.	Education	0.007	−0.026	0.028	1						
5.	Tenure	−0.093^*^	0.740^**^	−0.641^**^	−0.061	1					
6.	EL	−0.042	0.063	−0.141^**^	−0.078	0.147^**^	1				
7.	OI	−0.025	0.089	−0.103^*^	−0.026	0.163^**^	0.550^**^	1			
8.	NC	−0.023	−0.075	0.125^**^	0.094^*^	−0.164^**^	−0.407^**^	−0.261^**^	1		
9.	COD	−0.096^*^	0.004	−0.034	0.010	0.037	0.133^**^	0.127^**^	0.204^**^	1	
10.	CID	−0.094^*^	0.039	−0.026	0.039	0.037	0.168^**^	0.222^**^	0.081	0.516^**^	1
	Mean	0.472	1.882	1.465	2.991	2.004	4.004	3.950	2.729	3.344	3.367
	SD	0.500	0.760	0.499	0.609	0.961	0.547	0.670	0.817	0.757	0.797

MS, marital status; EL, ethical leadership; OI, organizational identification; NC, normative conflict; COD, constructive organizational deviance; CID, constructive interpersonal deviance; The same below

* *p* < 0.05;

** *p* < 0.01.

### Common method bias

In this research, anonymous questions and reverse items are used to control common method bias, but since all items are self-reported by a single respondent, common method variance is inevitable. In this study, the most commonly used Harman single factor test and Unmeasured Latent Method Construct (ULMC) test were used to test the possible common method bias. First of all, Harman single factor test’s result shows that the first factor explained 27.9%, below the recommended threshold of 40%, so there was no significant common method bias (Harman, 1976). However, as suggested by Podsakoff et al. (2003), although the Harman single factor test is widely used, it may be inadequate in detecting common method bias. Therefore, this study adopted ULMC to further detect the common method bias of this study (Hulland et al., 2018). The result shows that the variance extracted from the potential common method variance factor was 0.171, lower than the 0.25 threshold proposed by Williams et al. (1989). In summary, there is no serious common method bias in this study.

### Validity analysis

First, in this research, all the scales are mature scales published in foreign journals and verified by many empirical studies in the Chinese context, thus, the content validity was good. Second, the AVE and CR of all variables are calculated, and the results are shown in Table 3. The AVE of ethical leadership, normative conflict, organizational identification, COD and CID are 0.507, 0.556, 0.608, 0.544, 0.596, respectively, all exceed the recommended minimum guideline of AVE > 0.5; The CR of each variable are 0.911, 0.882, 0.925, 0.855, 0.854, respectively, all exceed the thresholds of CR > 0.7, therefore, the convergent validity is good. Third, Amos 26 is used to test the discriminant validity by confirmatory factor analysis. The results of confirmatory factor analysis in Table 4 show that the five-factor model has the best goodness of fit (NFI = 0.922, TLI = 0.915, CFI = 0.922, RMSEA = 0.055), indicating that the five constructs in this research have good discriminant validity.

**Table 3:** Reliability and validity (*N* = 449).

**Variables**	**Items**	**Factor loadings**	**Cronbach’α**	**AVE**	**CR**
EL	Listens to what employees have to say	0.670	0.905	0.507	0.911
	Disciplines employees who violate ethical standards	0.871			
	Conducts his/her personal life in an ethical manner	0.691			
	Has the best interests of employees in mind	0.723			
	Makes fair and balanced decisions	0.679			
	Can be trusted	0.732			
	Discusses business ethics or values with employees	0.672			
	Sets an example of how to do things the right way in terms of ethics	0.693			
	Defines success not just by results but also the way that they are obtained	0.664			
	When making decisions, asks “what is the right thing to do?”	0.700			
OI	When someone criticizes our company, it feels like a personal insult.	0.784	0.881	0.556	0.882
	I am very interested in what others think about our company	0.729			
	When I talk about my company, I usually say “we” rather than “they”	0.733			
	Company’s successes are my successes	0.725			
	When someone praises our company, it feels like a personal compliment	0.771			
	If a story in the media criticized our company, I would feel embarrassed	0.729			
NC	This company falls short of what it could be because of the rules and norms it enforces on employees	0.812	0.924	0.608	0.925
	This company could be so much better if it followed different rules or norms	0.792			
	This company will never reach its true potential until it changes its practices	0.769			
	The standards of this company encourage the wrong sort of behavior from employees	0.746			
	This company has rules or norms that lead to wasteful or counterproductive behavior	0.854			
	This company could be much more efficient if people could follow different rules or norms	0.801			
	The values of this company are not accurately reflected in the rules and norms it sets	0.745			
	I think that the rules and norms of this company are valid and reasonable(R)	0.710			
COD	Sought seek to bend or break the rules in order to perform your job	0.701	0.848	0.544	0.855
	Violated company procedures in order to solve a problem	0.720			
	Departed from organizational procedures to solve a customer’s problem	0.757			
	Bent a rule to satisfy a customer’s needs	0.630			
	Departed from dysfunctional organizational policies or procedures to solve a problem	0.859			
CID	Reported a wrong-doing to co-workers to bring about a positive organizational change	0.816	0.851	0.596	0.854
	Did not follow the orders of your supervisor in order to improve work procedures	0.794			
	Disagreed with others in your workgroup in order to improve the current work procedures	0.653			
	Disobeyed your supervisor’s instructions to perform more efficiently	0.812			

**Table 4:** Results of CFAs: comparison of measurement models.

**Models**	***χ*** ^**2**^	**df**	***χ*** ^**2**^ **/df**	**IFI**	**TLI**	**CFI**	**RMSEA**
Five-factor model (EL, NC, OI, COD, CID)	1135.261	485	2.341	0.922	0.915	0.922	0.055
Four-factor model (EL, NC, OI, COD + CID)	1545.321	489	3.160	0.874	0.863	0.873	0.069
Three-factor model (EL, NC + OI, COD + CID)	2767.962	492	5.626	0.728	0.707	0.727	0.102
Two-factor model (EL + NC + OI, COD + CID)	3895.759	494	7.886	0.594	0.564	0.592	0.124
One-factor model (EL + NC + OI + COD + CID)	5243.506	495	10.593	0.433	0.392	0.430	0.146

### Empirical results

**Figure 2:**
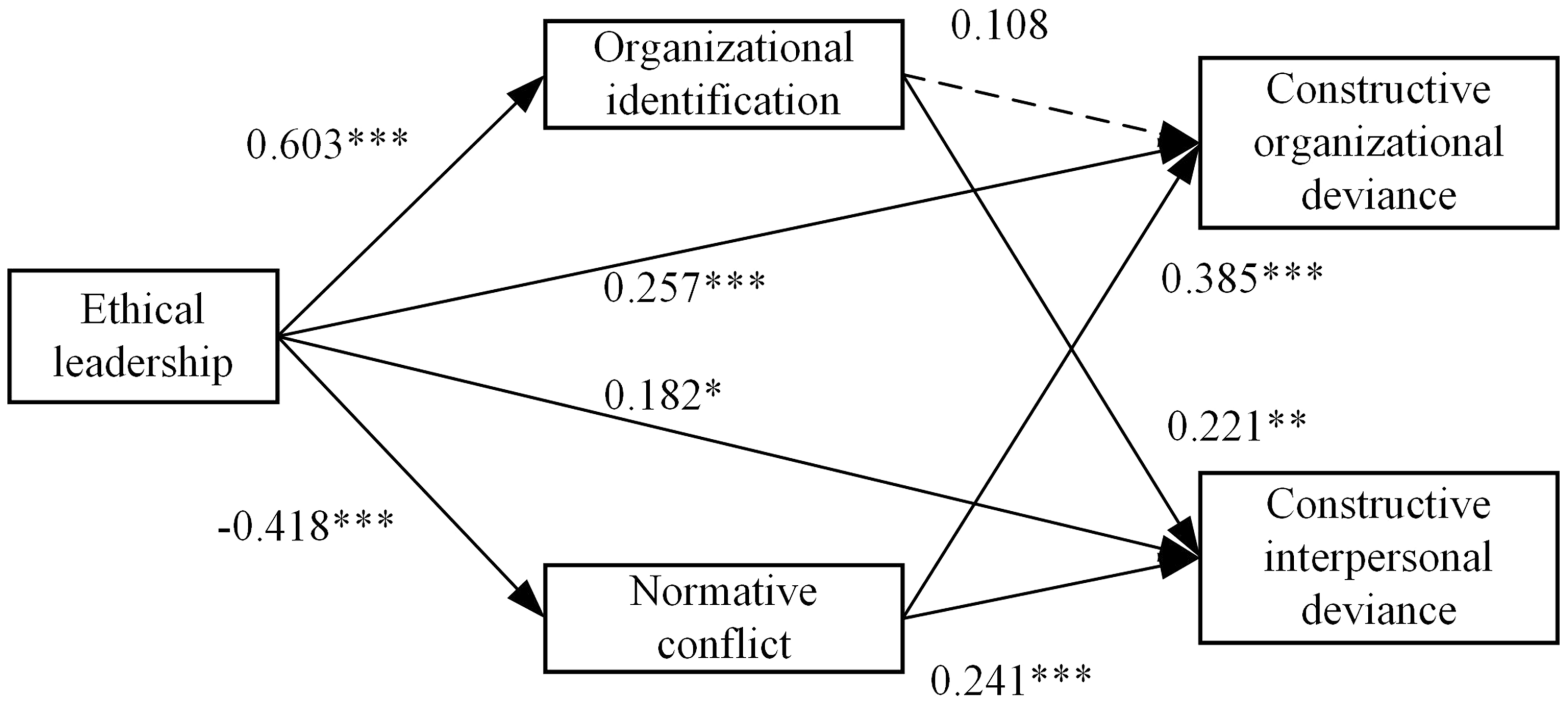
Path coefficients.

The Structural equation model was used to test the relationship between latent variables in this study. Specifically, this research used AMOS 26 and conducted 5,000 bootstrapping samplings to examine the path coefficients, mediating effects, and total effects among variables. The path coefficients are shown in Table 5 and Figure 2, and the results mediating effects and total effects are shown in Table 6. As shown in Table 6, the total effect of ethical leadership on constructive organizational deviance is 0.160, 95% bias-corrected confidence interval (CI) is [0.035, 0. 284], excluding 0, hypothesis 1 is supported. The total effect of ethical leadership on constructive interpersonal deviance is 0.214, and the 95% bias-corrected CI is [0.084, 0.337], excluding 0, so hypothesis 2 is supported. In addition, compared with CID, ethical leadership plays a weaker role in promoting COD, hypothesis 3 is supported.

**Table 5:** Path coefficients.

**Path**	**Estimate**	**SE**	**C.R.**	***P***	**Std.**
EL → OI	0.734	0.070	10.424	0.000	0.603
EL → NC	−0.622	0.081	−7.686	0.000	−0.418
EL → COD	0.305	0.088	3.472	0.000	0.257
EL → CID	0.274	0.111	2.468	0.014	0.182
OI → COD	0.105	0.066	1.594	0.111	0.108
OI → CID	0.273	0.086	3.185	0.001	0.221
NC → COD	0.308	0.049	6.253	0.000	0.385
NC → CID	0.244	0.059	4.109	0.000	0.241

**Table 6:** Standardized direct, indirect, total effects and 95% bias-corrected CI.

**Path**	**Estimate**	**SE**	**95% bias-corrected CI**
*Direct effect*			
EL → COD	0.257	0.083	[0.092, 0.420]
EL → CID	0.182	0.083	[0.014, 0.347]
*Indirect effect*			
EL → OI → COD	0.065	0.045	[−0.020, 0.156]
EL → OI → CID	0.133	0.046	[0.044, 0.227]
EL → NC → COD	−0.161	0.03	[−0.228, −0.110]
EL → NC → CID	−0.101	0.029	[−0.163, −0.047]
*Total effect*			
EL → COD	0.16	0.062	[0.035, 0.284]
EL → CID	0.214	0.064	[0.084, 0.337]

This study uses the same method to test the mediating role of organizational identification and normative conflict. As shown in Table 6, the mediating effect of the EL → OI → COD path is 0.065, and 95% bias-corrected CI is [−0.020, 0.156], including 0. Therefore, organizational identification has no significant mediating effect between ethical leadership and constructive organizational deviance, and hypothesis 4 is not supported. The mediating effect of the EL → OI → CID path is 0.133, 95% bias-corrected CI is [0.044, 0.227], excluding 0, hypothesis 5 is supported, and compared with CID, ethical leadership plays a weaker role in promoting COD through organizational identification, hypothesis 6 is supported. The mediating effect of the EL → NC → COD is −0.161, 95% bias-corrected CI is [−0.228, −0.110], excluding 0, hypothesis 7 is supported. The mediating effect of the EL → NC → CID is −0.101, 95% bias-corrected CI is [−0.163, −0.047], excluding 0, and hypothesis 8 is supported. Compared with CID, ethical leadership has a stronger inhibitive effect on COD through the normative conflict, hypothesis 9 is also supported.

## Discussion

This research discusses the double-edged sword effect of ethical leadership on constructive deviance, and responds to the previous researchers’ call to further explore the antecedents of constructive deviance from the perspective of ethics (Liu et al., 2020; Zhang et al., 2021). In addition, the research results of Zhang et al. (2021) show that leader moral humility is better than ethical leadership in promoting constructive deviance, but the mechanism of ethical leadership is not further discussed. The results of this study can provide some references, that is, although ethical leadership can promote the constructive intention of employees, it pays more attention to the normative standards in the organization than leader moral humility, which will reduce the normative conflict of employees and inhibit their constructive deviance.

In addition, this research examines the mediating role of organizational identification and normative conflict between ethical leadership and constructive deviance. However, the analysis results show that organizational identification has no significant mediating effect between ethical leadership and COD, which is inconsistent with hypothesis 4 of this research. However, such a result is not surprising. Traditional social identity studies believed that individuals with high organizational identification are usually the maintainers of organizational norms, and for them, the benefits brought by engaging in constructive deviance may be far less than the costs of breaking formal organizational norms. On the one hand, Blader et al. (2017) held that the effect of organizational identification on employee behavior would be influenced by the organizational environment and evaluation of organizational members (e.g., superiors, colleagues and subordinates). Ethical leadership values organizational norms, if employees break formal organizational norms may be seen as a public challenge to the authority of the leadership. In contrast, ethical leadership is willing to listen to reasonable suggestions from employees and encourage members to speak out freely. Ethical leadership does not care about or even advocate breaking interpersonal norms. In fact, the results show that organizational identification plays a significant mediating role between ethical leadership and CID. On the other hand, there may be a nonlinear relationship between organizational identification and constructive deviance. Shahjehan et al. (2020) found that there was a U-shaped relationship between organizational identification and defensive voice. Employees with high organizational identification voice for collective interests, while employees with low organizational identification voice for personal interests.

What is more, the negative mediating effect of normative conflict in this research is masked by the direct effect of ethical leadership on constructive deviance, but this does not mean that the mediating effect of normative conflict is worthless. First of all, as above, ethical leadership has a screening effect on constructive deviance, and can raise the threshold of the employee deviance by setting the violation cost and screen of irrational constructive deviance, thus improving the overall quality of staff constructive deviance, it conforms to the claims of the present study, namely the negative effect of ethical leadership on the constructive deviance of employees is actually a positive screening effect, rather than knocking down all of them. Secondly, Zhao et al. (2010) called the situation in which direct and indirect effects exist at the same time and the direction is opposite as competitive mediation, indicating that there are other positive effect paths between ethical leadership and constructive deviance, which can point out the direction for further research. For example, although constructive deviance is the pro-organization behavior out of altruistic motive, employees may also engage in constructive deviance out of selfish motives such as facilitating their work or winning the trust of superiors.

In summary, based on the normative conflict model, this study proposes a dual-pathway model that is constituted of organizational identification and normative conflict, and examines the double-edged sword effect of ethical leadership on subordinates’ constructive deviance. Specifically, ethical leadership stimulates employees’ constructive intention by improving their organizational identification and weakening employees’ normative conflict, restraining their deviant motivation, then realizing the double-edged sword effect on constructive deviance. In general, the overall effect of ethical leadership on employee constructive deviance is positive, and the positive effect of ethical leadership on CID is significantly higher than that of COD.

### Theoretical implications

First, this research examines the double-edged sword effect of ethical leadership on constructive deviance, providing a new idea for previous studies on the impact of ethical leadership on constructive deviance from a positive or negative perspective (Xu and Zhu, 2017; Gao et al., 2019). In recent years, more and more studies focus on the suppression effect of ethical leadership on extra-role behavior (Miao et al., 2013), but most only examine the ethical leadership’s “too much of a good thing” effect (Mo et al., 2019; Yam et al., 2019), there is little research including the positive and negative effect of ethical leadership in the same framework, which is bad for completely understanding the mechanism of ethical leadership on employee behavior. This research proposes that ethical leadership has two core attributes, ethics and compliance, and has independent positive and negative effects on constructive deviance, which provides a powerful supplement for the mechanism of ethical leadership’s influence on employee behavior.

Secondly, the normative conflict model is revised reasonably in this research. The core hypothesis of the normative conflict model is that normative conflict moderates the relationship between organizational identification and member dissent, that is, normative conflict is the boundary condition of the relationship between organizational identification and member dissent (Packer, 2008). This research further considers normative conflict as an important mechanism of ethical leadership influencing constructive deviance rather than just a boundary condition. On the one hand, normative conflict is as important an antecedent of constructive deviance as organizational identification. According to the normative conflict model, when group members experience high-level normative conflict, they will actively oppose group norms (Packer, 2008). Obviously, normative conflict is an important source of employee deviant motivation; On the other hand, normative conflict is malleable, and leaders can influence employees’ normative conflict objectively and subjectively by improving the rationality of organizational norms and emphasizing the authority of organizational norms. Therefore, based on the framework of the original normative conflict model, this research reconstructs the double-mediating model of organizational identification and normative conflict, and explores the double-edged sword effect of ethical leadership on constructive deviance.

Thirdly, this research confirms the explanatory power of the normative conflict model between leadership style and constructive deviance, and provides a new theoretical perspective for the formation mechanism of constructive deviance. In recent years, researchers have gradually begun to focus on the negative effects of constructive deviance, but most of them attribute the negative effects to the defects of constructive deviance, ignoring the quality problems caused by the behavior’s initiator with the low level of competence. To exert the effectiveness of constructive deviance, it is necessary not only to encourage employees to engage in constructive deviance, but also to screen out irrational and low-quality constructive deviance. On the one hand, the analysis results show that ethical leadership can stimulate the constructive motivation of employees by improving their organizational identification, and on the other hand, it can weaken the normative conflict of employees and inhibit their deviant motivation, so as to realize the double-edge sword effect on the constructive deviance. This conclusion is helpful to understand the formation mechanism of constructive deviance more comprehensively.

Finally, this research further explores the impact of ethical leadership on different types of constructive deviance. Compared with other positive leadership, ethical leadership pays more attention to employees’ compliance with organizational standards and normative expectations (Lemoine et al., 2019; Bush et al., 2020). Therefore, when exploring the relationship between ethical leadership and employee’s constructive deviance, it is necessary to consider the difference in the formal degree of norms, and study different types of constructive deviance separately. The data analysis results also support this view. Although both of them belong to constructive deviance, the promotion effect of ethical leadership on constructive organizational deviance that violates the formal norms of the organization is significantly weaker than that of constructive interpersonal deviance.

### Practical implications

Firstly, this study provides practical guidance for organizations to deal with the double-edged sword effects of constructive deviance. In most cases, constructive deviance helps to improve employees’ innovation and organizational competitiveness (Li and Wang, 2021; Zhang et al., 2021). However, in the actual organizational situation, employees are unable to make the most favorable judgment for the organization due to the lack of ability and knowledge, and some constructive deviance cannot achieve their expected effects, or even bring losses to the organization (Cui et al., 2020). Therefore, enterprises should not give free rein to employees while giving them discretion. They need to emphasize compliance with organizational normative expectations and raise the threshold for employees to engage in constructive deviance, thus playing a role in screening. Through such managerial measures, the overall quantity of constructive deviance within the organization decreases, but the overall quality improves significantly. However, organizations need to strike a balance between advocating norms and adhering to them, overemphasizing abiding by organizational norms may inhibit employees’ innovation and affect the long-term development of the organization.

Secondly, ethical leadership should not be regarded as the opposite of organizational innovation. In recent years, with the deeper excavation of ethical leadership, more and more scholars proposed that ethical leadership’s excessive attention on organizational norms will hinder the development of organizational innovation (Mo et al., 2019; Li and Wang, 2021). In fact, the results of this study show that normative conflict may be a barometer of the rationality of organizational norms. The occurrence of employees’ constructive deviance and creative deviance represents that there is room for improvement in the existing organizational norms. Therefore, ethical leadership may not be a stubborn guardian of rules, the purpose of ethical leadership is shaping the best organizational norms so that employees can conduct innovative behaviors without violating organizational norms. This study suggests that organizations can recruit and cultivate ethical leadership using human resource management measures, because ethical leadership may not stand on the opposite side of organizational innovation, but promote efficient organizational innovation.

Finally, this study suggests that organizations should pay attention to the cultivation of employees’ organizational identification, and give appropriate guidance according to the actual situation of the organization. Employees with high organizational identification do not always follow regulations, and sometimes choose to break organizational norms for the benefit of the organization (Packer, 2008; Dahling and Gutworth, 2017), some employees even engage in unethical pro-organizational behavior for the benefit of the organization, which is obviously not conducive to the long-term development of the organization (Naseer et al., 2020). What is more, different types of organizations have different requirements for their employees. For example, managers of innovative organizations need to cultivate an ends-focused atmosphere to stimulate employees’ organizational-welfare motivation, while organizations such as the military place more emphasis on the procedures and methods, and need to form a means-focused atmosphere inside the organization to stimulate the affiliative motivation of organization members (Blader et al., 2017). Therefore, in management practice, organization managers not only need to cultivate the organizational identification of organization members, but also need to shape correct values for employees, and give appropriate guidance to employees according to the specific situation and cultural background of the organization.

### Limitations and future directions

First of all, this study is a cross-sectional study in nature and cannot deduce the causal relationship between the variables studied. Therefore, future studies can conduct experimental study confirm the causal direction of the proposed model and test the robustness of the conclusions in this study. In addition, future research can also adopt qualitative research, such as case analysis, to further verify the double-edged sword effect of ethical leadership on constructive deviance in practical situations.

Secondly, this study only proposes the mechanism of ethical leadership on constructive deviance, and does not further explore the boundary conditions of this model. Although the results of this study show that the formality of norms may be an important factor influencing the relationship between ethical leadership and constructive deviance. However, it is necessary to further explore the boundary conditions of this model. For example, in the context of high collectivism culture like China, ethical leadership’s compliance essence may be further amplified (Wang et al., 2022). So future research can verify the moderating effect of Chinese cultural content (such as Chinese traditionality) on the proposed model, and explore whether there are significant differences in different cultural backgrounds.

Third, this study only examines the mediating role of organizational identification and normative conflict between ethical leadership and constructive deviance. However, the significant main effect in the analysis results suggests that there may be other mediating mechanisms between ethical leadership and constructive deviance, the future study can excavate potential mediation mechanisms in light of other theoretical perspectives.

Finally, this study theoretically analyzes the double-edged sword effect of ethical leadership on constructive deviance, which can control the number and improve the quality of constructive deviance in the organization, but the research results can only prove the inhibition path of ethical leadership on constructive deviance, and cannot reflect the improvement of its quality. Therefore, future research can use the combination of self-evaluation and superior evaluation to measure employees’ constructive deviance, and take the difference between them as the basis for judging the quality of constructive deviance.

## Data availability statement

The raw data supporting the conclusions of this article will be made available by the authors, without undue reservation.

## Ethics statement

The studies involving human participants were reviewed and approved by Ethics Committee of Liaoning Technical University. The patients/participants provided their written informed consent to participate in this study.

## Author contributions

All authors listed have made a substantial, direct, and intellectual contribution to the work and approved it for publication.

## Funding

This study was supported by the National Natural Science Foundation of China (nos. 52174184 and 51504126), the Department of Education of Liaoning Province (no. LJ2020JCW002), the Department of Education of Liaoning Province (no. L20BGL030), and the Humanities and Social Science Foundation of Ministry of Education of the People’s Republic of China (no. 19YJA630038).

## Conflict of interest

The authors declare that the research was conducted in the absence of any commercial or financial relationships that could be construed as a potential conflict of interest.

## Publisher’s note

All claims expressed in this article are solely those of the authors and do not necessarily represent those of their affiliated organizations, or those of the publisher, the editors and the reviewers. Any product that may be evaluated in this article, or claim that may be made by its manufacturer, is not guaranteed or endorsed by the publisher.
